# Weakly interacting species as drivers of ecological stability

**DOI:** 10.1098/rspb.2025.0604

**Published:** 2025-06-11

**Authors:** Deirdre McClean, Alain Finn, Ian Donohue

**Affiliations:** ^1^ Institute of Ecology and Evolution, University of Edinburgh School of Biological Sciences, Edinburgh EH9 3FL, UK; ^2^ Department of Biological Sciences, National University of Ireland Maynooth, Maynooth W23 F2H6, Ireland; ^3^ School of Natural Sciences, Trinity College Dublin, Dublin D02 PN40, Ireland

**Keywords:** ecology, stability, community ecology, microcosm, experiment, species interactions

## Abstract

Determining how individual species can act to moderate the stability of entire ecosystems is a pressing challenge in a world undergoing rapid environmental change. Here, we show that even very weakly interacting species with no discernible effect on ecological dynamics can contribute substantially to ecosystem stability. Further, the nature of this contribution depends on biotic context, and both the type and complexity of interspecific interactions in the community. By manipulating multitrophic aquatic microcosm communities experimentally, we found that the contributions of a bacteriophage parasite to overall system stability following a pulse perturbation were variously stabilizing, destabilizing and neutral, depending on the presence of competitor or predator species of its bacterial host. This was despite the phage itself having no detectable effect on the biomass or growth rates of its host. Our results demonstrate the pivotal importance of both weak and indirect interactions in moderating the stability of whole ecological networks, and have profound implications for our ability to predict the consequences of perturbations on ecosystems.

## 1. Introduction

The collective dynamics of species and their responses to disturbance ultimately govern ecosystem stability [1–5]. Community dynamics are, however, determined not only by the identity of their constituent species and the size of their populations but also by the network of interactions in which they are embedded [6–10]. Individuals respond to their local environment and immediate pressures, which include a multitude of biotic interactions such as competition, predation and parasitism. Such interactions may be direct, such as the impact of a parasite on its host, or indirect, such as the impact of a parasite on a predator or prey of its host (e.g. [11,12]). Species loss or the invasion of new species creates changes in interaction networks that can lead to propagation of indirect effects that cascade through the system [13–17]. Understanding the role that individual species play in shaping the stability and functioning of communities thus requires an understanding of both the nature and the complexity of interaction networks within which those species are nested.

**Figure 1.**
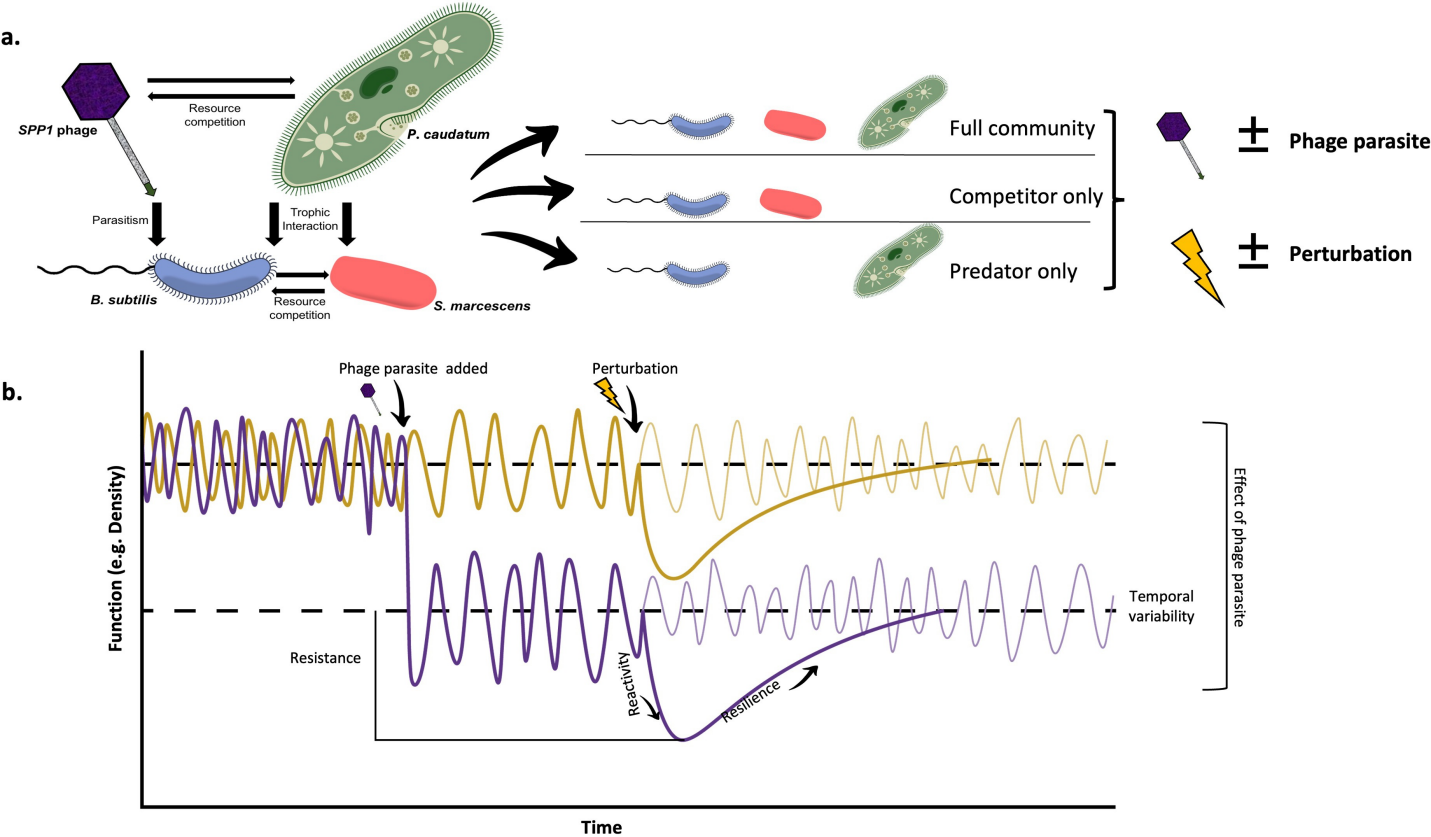
Our microcosm food webs and experimental design. (a) Our experimental treatments consisted of communities comprising host bacteria *Bacillus subtilis* (in blue), in combination with either competitor bacterium *Serratia marcescens* (red), the predatory protist *Paramecium caudatum* (green) or both together. Specialist parasitic bacteriophage of *B. subtilis*, SPP1 phage, was added to half of microcosms in each community treatment and microcosms were assigned to perturbed or unperturbed treatments in a factorial design, with each of our 12 experimental treatments replicated seven times. (b) We quantified the contribution of SPP1 phage to the resistance, reactivity, resilience and temporal variability of communities (see table 1 for a detailed description of stability components and their quantification) by comparing stability responses in communities in which the phage was present (purple lines) and those in which it was not (yellow lines) through comparison of perturbed (bold lines) with equivalent unperturbed (faded lines) treatments. If a measure of stability was increased in the absence of the phage compared with when it was present, this implies that the phage contributes negatively to this measure of stability, and *vice versa*.

Ecological stability is a multidimensional concept [4,18–20] encompassing many components that together capture the dynamics of communities and their responses to disturbance. These components include the variability, resistance, resilience and reactivity of communities (figure 1; see table 1 for a detailed description of these stability components). These various stability facets may behave largely independently of one another, or be tightly interdependent, depending on the environment, the nature of the disturbance and the composition of the community [4,18,19,22,23]. This means that simultaneous measurement of multiple components of stability is essential to fully understand the overall impacts of disturbances on ecosystems [18].

**Table 1.** Components of ecological stability measured in this study; their definition, quantification and interpretation were based largely upon Pimm [21], Donohue *et al*. [4], Hillebrand *et al*. [19] and White *et al*. [5]. We then measured the contribution of our focal bacteriophage to the stability of the community as the effect size (log response ratio) of stability responses in the presence of the phage compared with those in its absence from the otherwise equivalent community for each component of stability ([5,22]; figure 1b; see §2).

component of stability	method of quantification	interpretation
resistance	the maximum log response ratio of total community density in perturbed relative to equivalent unperturbed treatments	the extent of density change in response to perturbation. The nature of our experimental pulse perturbation always resulted in reductions in the densities of every species in our microcosms. Large negative values indicate large reductions in density following perturbation and, therefore, low resistance
reactivity	slope of linear regression between points of log response ratio over time immediately following perturbation until point of maximum deviation of perturbed from unperturbed treatments	increasingly negative values indicate increasingly reactive systems and, thus, lower stability. Increasing positive values correspond to a lack of reactivity and, therefore, enhanced stability
resilience	slope of linear regression of log response ratio over time from the point of maximum displacement between perturbed and unperturbed treatments until the point of recovery	increasingly positive values correspond to higher resilience (and stability); increasingly negative values indicate further deviation from unperturbed plots (i.e. low resilience and stability)
temporal variability	the coefficient of variance (CV; that is, standard deviation divided by the mean) of total density within experimental microcosms over time, measured from day 5 until the end of the experiment. Detrended to remove potentially confounding effects of biomass change over the duration of the experiment [3]. As it does not require explicit perturbation in order to be quantified, we measured temporal variability from unperturbed microcosms only	high values correspond to greater temporal variability and, thus, lower stability

The loss or addition of species can cause dramatic changes in the structure and stability of whole communities [15,24–26], the extent of which may be determined by whether this species is considered to be a strong or weak interactor [13,14,16,27]. Strongly interacting species—those species with a large *per capita* effect on the density of other species in the community—are considered to be particularly important drivers of community dynamics (e.g. [28–30]). However, relatively weakly interacting species can also play a crucial role in determining community stability through, for example, dampening oscillatory predator–prey or competitor interactions (e.g. [16,31]). What has been less well studied is the overall contribution of individual species to the stability of whole communities and, crucially, the consistency of those contributions in light of the biotic environment in which a species finds itself [5,22]. Does a given species always contribute to the stability of the system in a consistent manner, or does the context of the biological interaction network in which it finds itself alter its impact?

Here, we test the extent to which the contribution of individual species to community stability is moderated by the nature and complexity of interspecific interactions within ecological networks. We manipulated multitrophic aquatic microbial communities of varying complexity experimentally, focusing particularly on the role of a lytic bacteriophage parasite in shaping the overall community response to perturbation. Even though parasites are key contributors to biodiversity in every ecosystem throughout the biosphere [12,32], understanding of their contribution to ecological dynamics, functioning and stability remains remarkably poor [11,12,33–35].

Bacteriophages (phages) are obligate intracellular parasites that infect bacteria populations in every environment that they occupy. Owing to their relative ease of culturing and long history of use in laboratory conditions, many bacteriophages present themselves as ideal candidates for use in microcosm studies. Our focal bacteriophage (SPP1 bacteriophage, a specialist lytic phage parasite of *Bacillus subtilis* [36]) has only one direct interspecific interaction in our experimental system—that with its bacterial host *B. subtilis*. As this is a lytic phage, the individual interaction is strong—when a bacterium is infected it is killed—though it will not have a direct effect on any of the other species in the community. Here, we benchmark the effects of the SPP1 bacteriophage on its host (*B. subtilis*), competitor bacterium *Serratia marcescens*, and a natural generalist predator of bacteria, *Paramecium caudatum*. We assessed the contribution of the bacteriophage to four components of community stability in each of our experimental communities (that is, their temporal variability, reactivity, resistance and resilience to a pulse perturbation; figure 1 and table 1). We combined this with tests of the direct effects of the bacteriophage on its host’s growth and competitive ability. This experimental framework allowed us to directly assess the relative impacts of direct and indirect species interactions on the wider community dynamics and stability.

## 2. Material and methods

### (a) Experimental design

Our experimental design combined the presence and absence of our focal SPP1 bacteriophage in a factorial design with three sets of species combinations of its host species *B. subtilis*, together with: (i) *S. marcescens* (a competitor of *B. subtilis*) in isolation; (ii) *P. caudatum* (a polyphagous predator of *B. subtilis*) in isolation; and (iii) both *S. marcescens* and *Paramecium* in combination. Each of our six sets of species combinations was also exposed to one of two levels of disturbance (that is, perturbed and unperturbed), resulting in a total of 12 experimental treatments (figure 1a), each replicated seven times. Our experimental pulse perturbation took the form of a mass mortality event, where the density of all species in the system was reduced by approximately 90% [37,38]. The pulse perturbation was applied 4 days after the commencement of the experiment after the microcosm communities were first allowed to equilibrate following their initial assembly.

### (b) Community assembly

Culture methods followed closely those detailed in McClean *et al*. [38,39] and Leary *et al.* [40]. Microcosms consisted of loosely capped 200 ml glass bottles with 15 g glass microbeads providing habitat structure, allowing the organisms to interact as they would in a more naturally complex environment. Each microcosm received 100 ml medium consisting of one protist pellet (Carolina Biological Supply, Burlington, NC, USA) per 1 l spring water, and two wheat seeds to provide a slow-release nutrient source. All media were sterilized before use. Microcosms were maintained at 22°C under a 12 : 12 h light : dark cycle. Nutrients in the microcosms were replenished with weekly replacement of 7 ml of the microcosm volume with sterile medium and one additional sterile wheat seed. *Paramecium* species were obtained from Blades Biological UK. Bacteria strains and SPP1 phage were taken from laboratory stock cultures. The *Paramecium* protist cultures used for this experiment were laboratory cultures and, therefore, while not inoculated with any bacterial populations, were not entirely sterile. To account for this, *Paramecium* was washed with sterile medium before addition to the microcosms to minimize contamination. The remaining medium from the washing process (minus *Paramecium*) was combined and mixed thoroughly, and a similar volume to the protist treatments was then added to all microcosms to ensure that any bacteria present in the medium had an equal chance of colonizing each microcosm in every treatment. We identified one contaminant bacterial species, *Klebsiella*, that colonized all microcosm units in this manner and was therefore treated as a standard ‘background’ member of each community and analysed alongside the other species. No other contaminant species were recorded in any of our experimental microcosms.

Overnight cultures of strains NCIB3610 (*B. subtilis*) and *S. marcescens* ATCC 29632, grown in tryptone yeast (TY) medium (Luria Burtani broth supplemented with 10 mM MgSO_4_ and 100 mM MnSO_4_ after autoclaving [41]), were diluted into fresh TY medium at optical density (OD)_600_ *ca* 0.03 and grown at 37°C until late exponential phase (OD_600_ *ca* 1.0), at which time 1 ml of each bacterial culture was inoculated into 100 ml microcosm medium, as required for experimental treatments. Microcosms were left for 24 h at 37°C to facilitate the growth of the bacteria prior to the addition of the SPP1 phage. To each microcosm, 1 ml of phage stock solution (1.7 × 10^4^ pfu ml^−1^ diluted to 10^−3^ in sterile phosphate-buffered saline (PBS)) was added as required. These concentrations were chosen based on the results of pilot experiments, where we aimed to find a balance between having a sufficiently high density of phage to ensure that it would be consistently detectable, but would not overwhelm the bacteria in our microcosms. Microcosms were then left for a further 24 h at 37°C to facilitate the growth of the bacteria and ensure sufficient numbers before the addition of the generalist bacterivore *Paramecium* as required for experimental treatments. Microcosms with *P. caudatum* (approx. 50–70 individuals) were allowed to settle for 2 days at 22°C after inoculation. Microcosms allocated to perturbed treatments were swirled to homogenize communities, and 90% (90 ml) microcosm medium was removed on the fourth day of the experiment. The microcosms were then refilled with 90 ml sterilized medium.

### (c) Community monitoring

The point of addition of the predator *Paramecium* was considered as day 0 of the experiment. The experiment lasted 10 days. Samples of bacteria, phage and protists were taken daily for the duration of the experiment. Protists were sampled by gently swirling the microcosms to homogenize contents and to suspend the protists. A 0.1 ml sample was then examined using stereo (Olympus SZX9) and compound (Olympus BX60) microscopes. Bacterial density was measured through direct colony counts on plates from appropriately diluted samples. Phage numbers were measured through direct plaque counts on plates from appropriately diluted samples. Prior to plating, 3 ml of standard TY medium was inoculated with *B. subtilis* and incubated at 37°C for a minimum of 4 h or until an OD_600_ of 0.9–1.0 was achieved. Plates were poured with TY agar (1.5%) and allowed to set. Two hundred microlitres of the *B. subtilis* culture was added to 10 ml centrifuge tubes followed by 200 µl of the microcosm sample. This was then mixed gently by hand and incubated at 37°C for 15 min. Post incubation, 3 ml of soft agar (0.5%) was then added to each tube, swirled and then poured onto the pre-set 1.5% plates and incubated overnight at 37°C. The number of plaques on each plate was then counted.

### (d) Growth analysis and competitive ability

Bacterial growth curves indicate how well bacteria are adapted to their growing environment and whether there are strong physiological differences in growth dynamics among populations exposed to different conditions. We isolated eight colonies of *B. subtilis* from each microcosm on the final day of the experiment and measured their growth to quantify effects of SPP1 bacteriophage and our experimental perturbation and to test whether populations from the different ecological contexts differed in their adaptation to the abiotic conditions of the growth medium. We measured differences in three bacterial growth parameters—maximum growth density, lag time and growth rate—in evolved *B. subtilis* isolates at 10 min intervals over a 15 h period in the microcosm medium to assess differences in growth strategy among our experimental treatments.

We assessed the competitive ability of *B. subtilis* isolates from each mesocosm against the ancestral competitor *S. marcescens* as the extent of deviation from an initial 50 : 50 co-culturing ratio in 96-well plates containing 200 µl of microcosm medium over a 24 h period at 22°C. This was accomplished through counting proportions of the two bacteria based on differences in colony morphologies.

### (e) Data analyses

We quantified four components of functional stability (that is, based on measurements of total community density; [19]) for each microcosm community: their temporal variability, resistance, resilience and reactivity to our experimental pulse perturbation (figure 1b and table 1). We measured the effect of the disturbance on the community as the log response ratio. We then measured the contribution of our focal bacteriophage to the stability of the community as the effect size (log response ratio) of stability responses in the presence of the phage compared with those in its absence from the otherwise equivalent community for each component of stability ([5]; figure 1b). For this second log response ratio, owing to the presence of negative values in some cases, where necessary, we used a log(*x *+ *a*) transformation, where the minimum value when the constant *a* was added was 1 before the transformation. All analyses were carried out in R (v. 3.5.2; [42]).

We used permutational multivariate analysis of variance (PERMANOVA) to test whether the presence of the phage and/or our experimental perturbation affected the overall multivariate structure of the microcosm communities. All densities were log(*x *+ 1)-transformed prior to analysis to reduce potential bias caused by different population sizes. These analyses used data from days 5–10 of the experiment as the experimental perturbation took place on experimental day 4. Microcosm number and experimental day were incorporated as random factors. We used similarity percentages (SIMPER) analyses [43] to identify the contribution of each species in microcosm communities to the Bray–Curtis dissimilarity between phage and perturbed treatments. We used repeated measures analysis of variance (ANOVA) to test the impact of the presence of the phage and the perturbation on densities of *B. subtilis* in each of our experimental treatments. ANOVA was used to test the impact of phage presence and perturbation on each of our focal growth metrics and the competitive ability of *B. subtilis* populations isolated from microcosms at the end of the experiment.

## 3. Results 

**Figure 2.**
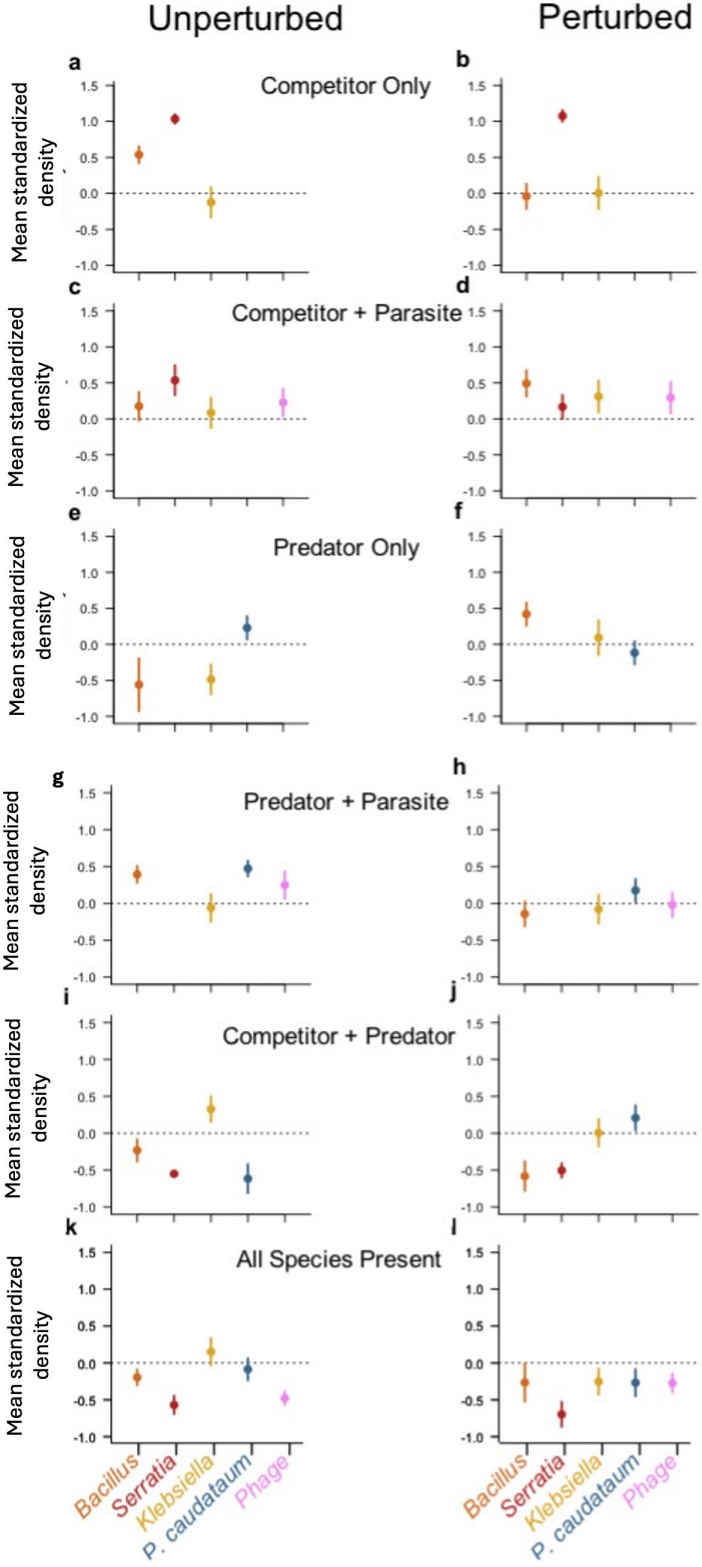
Normalized (mean standardized; overall mean across treatments represented by dashed line) densities (mean ± s.e.m., *n* = 7) of each of *Bacillus subtilis* (host species), *Serratia marcescens* (competitor), *Klebsiella*, *Paramecium caudatum* (predator) and our focal SPP1 phage in our experimental treatments in which *S. marcescens* was present (a–d,i,j), *P. caudatum* was present (e–j), and all species were present (k,l) in both unperturbed (left column) and perturbed (right column) communities. Shown are the combined data from experimental days 5−10, following the perturbation on experimental day 4. Data for individual populations over time are shown in electronic supplementary material, figures S1–S3.

The presence of the parasitic SPP1 bacteriophage moderated how the whole microbial community was affected by our experimental pulse perturbation (PERMANOVA, $F{\;}_{1,164}=3.91$, phage-perturbation interaction: *p* = 0.01; figure 2; electronic supplementary material, figures S1–S3). By comparing panels in figure 2 as an overview of the experiment, we can see the impact of the phage on the perturbation response for given populations. For example, when the phage was absent from the most complex communities containing competitor and predator species (figure 2i,j), it can be seen that in perturbed communities there was a small decrease in *B. subtilis* and *Klebsiella* populations, alongside an increase in *P. caudatum* densities. In contrast, when the phage was present in these communities (figure 2k,l), there was little change in *B. subtilis* densities, and a small decrease in those of both *Klebsiella* and *P. caudatum*. Similar comparisons can be made between the unperturbed and perturbed panels with and without phage for the smaller community treatments. Overall, the community perturbation response in the absence of the phage was driven primarily by changes in the density of the host’s competitor bacterium *S. marcesens* (SIMPER analysis, electronic supplementary material, table S1 and figures S1–S3). In communities containing the phage, however, the community perturbation response was driven primarily by changes in the density of the predatory *P. caudatum* (SIMPER analysis, electronic supplementary material, table S1 and figures S1–S3).

**Figure 3.**
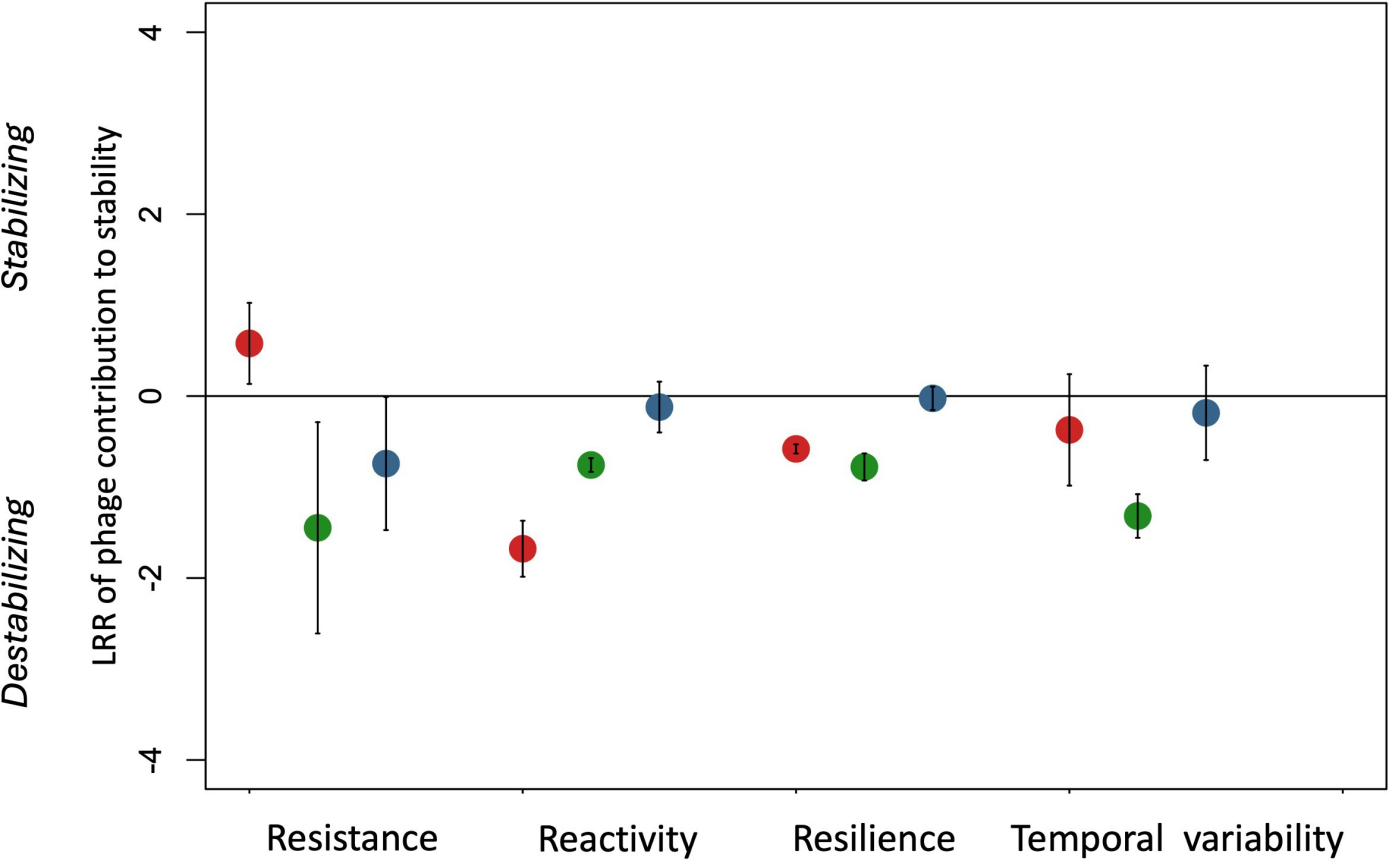
Phage contributions to multiple components of ecological stability (mean log response ratio (LRR) ± 95% CI; see table 1 for a description of stability components and their quantification and interpretation; the metrics here were log (*x *+ *a*)-transformed, where *a* was a constant such that when added the minimal value was 1) in microcosm communities containing competition with *Serratia marcescens* (red dots) or predation by *Paramecium caudatum* (green dots) and in complex communities containing both competition and predation (blue dots).

The presence of our focal phage species simultaneously stabilized and destabilized the microcosm communities. Whether the phage acted as a stabilizing or destabilizing force depended on the focal dimension of stability, and the nature and complexity of interspecific interactions in the system (figure 3 and table 1; electronic supplementary material, figure S4). Specifically, the phage contributed negatively to all dimensions of stability (that is, universally destabilized the system) in the presence of the predatory *P. caudatum* (green points in figure 3). However, it stabilized the system by increasing community resistance to our experimental perturbation when its host was exposed to competition with *S. marcescens* in the absence of *P. caudatum*, while simultaneously reducing community reactivity and resilience to perturbation (red points in figure 3). In contrast, the contributions of the phage to stability were consistently more muted as community complexity increased (blue points in figure 3), and it did not contribute significantly to any dimension of stability when both competitors and predators were present in the community.

The parasitic SPP1 bacteriophage contributed significantly to all of our measured dimensions of community stability in some way (figure 3). Remarkably, however, its presence did not affect the density of its host *B. subtilis* in any of our experimental communities (repeated measures ANOVA, *$F{\;}_{1,80}=1.07$*, *p* = 0.3, electronic supplementary material, figures S1–S3). Moreover, neither did it modify the perturbation response (that is, the difference in density between perturbed and unperturbed communities) of *B. subtilis* (repeated measures ANOVA, phage–perturbation interaction: *$F{\;}_{1,80}=2.5$*, *p* = 0.12, electronic supplementary material, figures S1–S3).

In order to more deeply understand the effect of the bacteriophage on its host and examine whether this effect varied between perturbed and unperturbed communities, we measured the growth rate, lag time and maximum density of *B. subtilis* from each treatment isolated at the end of the experiment (see Methods). We found that the presence of the phage did not alter the growth rate, the maximum density or the lag time of *B. subtilis* growth (electronic supplementary material, table S2). In contrast, lag time was extended in perturbed *B. subtilis* populations irrespective of phage infection, though the perturbation had no effect on *B. subtilis* growth rates (electronic supplementary material, table S2).

**Figure 4.**
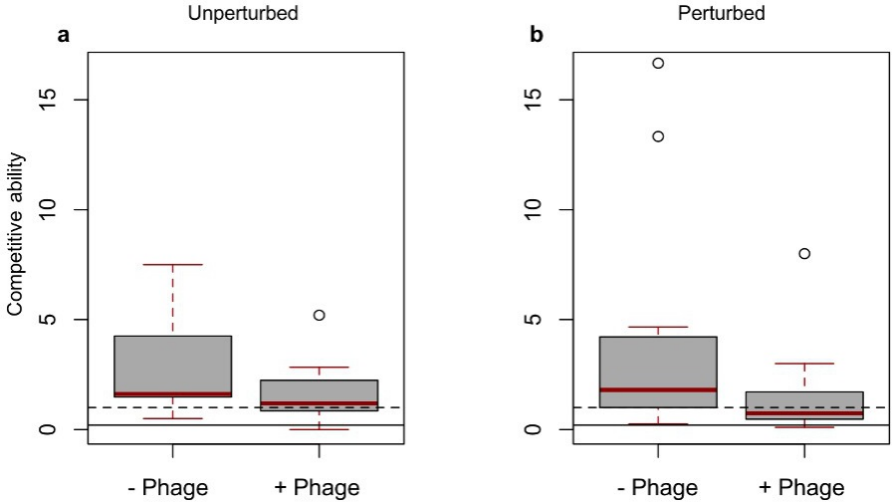
Competitive ability of *Bacillus subtilis* isolates, measured as the ratio of *B. subtilis* to *Serratia marcescens* (dashed line indicates a 1 : 1 ratio, solid line represents ancestral competitive ability) in both the presence and absence of the bacteriophage parasite in (a) unperturbed and (b) perturbed communities.

While neither our experimental perturbation nor the presence of the phage altered the maximum density of *B. subtilis* when present in isolation (ANOVA; perturbation: *$F{\;}_{1,74}=1.76$*, *p* = 0.19; phage: $F{\;}_{1,74}=0.16$, *p* = 0.19), their combined presence led to a reduced maximum density of *B. subtilis* (ANOVA; perturbation–phage interaction: *$F{\;}_{1,74}=3.9$*, *p* = 0.05). This suggests that *B. subtilis* exposed to the phage in perturbed environments had reduced competitive ability. We then tested this by assessing the competitive ability of evolved *B. subtilis* isolates from all treatments containing *S. marcescens* at the end of the experiment. We found that, though perturbation had no clear impact on the competitive ability of *B. subtilis* (ANOVA; main effect of perturbation: *$F{\;}_{1,65}=0.2$*, *p* = 0.68), isolates that had come from treatments containing the parasitic bacteriophage had reduced competitive ability relative to those where the phage was absent (ANOVA; main effect of phage: $F_{1,65}=7.24$, *p* = 0.009; figure 4). This was particularly evident in perturbed communities, where the presence of the phage was associated with a greater reduction in competitive ability than in unperturbed communities (Tukey HSD *p* < 0.05; figure 4).

## 4. Discussion

Our results show that even very weakly interacting species can moderate the effect of disturbances on ecosystems through their indirect interactions with other species in the community. Moreover, the nature of their contributions to the overall stability of the community depends on the biotic context in which the species finds itself. The contributions of our focal phage to community stability were frequently substantial, and occurred even though we found no impact of our experimental perturbation or community composition on the density of the phage, nor did we detect an impact of the phage on the perturbation response of their host in terms of density or growth rates. While this does not mean that the phage had no impact on the host, as its persistence throughout the experiment across all microcosms implies that the phage population was successfully killing its hosts, what these results highlight is that the effects and importance of weakly interacting species, in their direct effects on the densities of other species, may frequently go undetected and be underappreciated in standard ecological characterizations of species dynamics.

We found that the phage had both stabilizing and destabilizing contributions to the stability of the system in different ecological contexts. This result emphasizes the importance of taking a multidimensional approach to the characterization and quantification of ecological stability [4,18,19,44,45]. Within this framework, the contributions of any given species can be comprehensively assessed in relation to their impact on the stability of the wider community [5,22]. This allows a much more nuanced understanding of community interactions when undergoing disturbance, and provides a basis for a more targeted response when seeking to manage communities under stress.

Our data suggest that the presence of our focal phage species modified both the competitive ability of their hosts and aspects of their growth behaviour under disturbed conditions. This led, in turn, to knock-on consequences for the other species in the community. These results show, therefore, that even very weakly interacting species can dampen the effects of strong trophic or competitive interactions and have indirect effects that can cascade through ecological networks and modify responses of entire communities to disturbances. These effects became more muted in the most complex experimental communities we examined, where combined interactions appear to have dampened the influence of the phage on community dynamics.

Though the phage had no detectable effect on the dynamics or growth rates of its bacterial host under undisturbed conditions, the competitive ability of host *B. subtilis* was nonetheless reduced in the presence of the phage. This finding provides important insights into the likely mechanisms underpinning the indirect effects of the phage on community dynamics. SPP1 phage generally attacks the poles of the cell and the flagella of the bacterial host [46]. To best evade infection, resistant bacterial cells are, therefore, more likely to have fewer phage attack sites (flagella), leading to reduced motility [47,48]. Though we did not test directly the mechanisms of how the phage reduced the competitive ability of its host *B. subtilis*, our results suggest that the likely reduced motility of *B. subtilis* populations exposed to the phage contributed to their reduced competitive ability against ancestral competitors. Phages reduced the competitive ability of their hosts in all community contexts. This effect was, however, magnified in perturbed communities in particular (figure 4). Consistent with this, the densities of both competitor bacteria *S. marcescens* and *Klebsiella* increased in the presence of the phage. Taken together, these results suggest that the advantage for the host provided by phage defence may be contingent on the presence of the phage, and when this pressure is removed, the adapted bacteria are less able to compete with other species in their community. This would explain the loss of competitive advantage in *S. marcescens* with the addition of other species. As we were testing specifically for the impact of the phage on the community as a whole and its host bacterium, we did not explore the mechanisms by which *S. marcesen*s or *Klebsiella* may modify their interactions in the presence of one another or the predator and what implications this may have on the other members of the community.

Phage densities, while varying among communities, were quite low in all microcosms (figure 3). This, combined with a relatively low-nutrient medium [49], may explain why, despite encompassing numerous generations, we did not see a strong signal of the phage becoming fixed in the *B. subtilis* populations. Had we increased the density of phage populations we likely would have observed much stronger oscillatory dynamics between *B. subtilis* and the phage, possibly leading to one of the populations becoming extinct, and driving even stronger selection for phage resistance in the host bacterium.

Microcosms have become an increasingly valuable tool in ecological and evolutionary research owing to their ability to house complex communities in a small space and the relatively short time scales needed to conduct ecological experiments [27,50–53]. Using the bacteriophage host–parasite model has, therefore, huge potential for improving understanding of the role that parasites play in food web dynamics and, crucially, in understanding host response to multiple stressors. While our study is limited by the scale and context of species in our microbial experimental system, it nonetheless provides otherwise elusive mechanistic insights into interspecific interactions in complex multitrophic communities. Natural communities are inherently much more complex than the experimental communities we can establish in a laboratory. Through multispecies multitrophic microcosm studies such as this, however, we can disentangle individual interactions and isolate the impacts of single species on different communities simultaneously over relatively long biological time scales (that is, over many generations).

Our findings show that biotic context is crucial to consider for all species, and their interaction networks, when examining the impacts of disturbances on ecosystems. In particular, our findings highlight the fundamental importance of weak and indirect interactions in moderating the responses of entire communities to disturbance. Weak interactions may be difficult to detect in many systems, but our results underscore their potentially pivotal influence on the dynamics of the entire community. We conclude that predicting the responses of species and communities to disturbance on a rapidly changing planet presents a profound challenge to ecologists, requiring understanding not only of the roles played by individual species but also how their interspecific interaction dynamics vary across different ecological contexts.

## Data Availability

All data and code necessary to replicate our analysis can be found in Dryad [54]. Supplementary material is available online [55].
